# Assessing the Effect of Musical Congruency on Wine Tasting in a Live Performance Setting

**DOI:** 10.1177/2041669515593027

**Published:** 2015-07-30

**Authors:** Qian (Janice) Wang, Charles Spence

**Affiliations:** Department of Experimental Psychology, Oxford University, UK; Department of Experimental Psychology, Oxford University, UK

**Keywords:** crossmodal correspondences, music, taste, wine

## Abstract

At a wine tasting event with live classical music, we assessed whether participants would agree that certain wine and music pairings were congruent. We also assessed the effect of musical congruency on the wine tasting experience. The participants were given two wines to taste and two pieces of music—one chosen to match each wine—were performed live. Half of the participants tasted the wines while listening to the putatively more congruent music, the rest tasted the wines while listening to the putatively less congruent music. The participants rated the wine–music match and assessed the fruitiness, acidity, tannins, richness, complexity, length, and pleasantness of the wines. The results revealed that the music chosen to be congruent with each wine was indeed rated as a better match than the other piece of music. Furthermore, the music playing in the background also had a significant effect on the perceived acidity and fruitiness of the wines. These findings therefore provide further support for the view that music can modify the wine drinking experience. However, the present results leave open the question of whether the crossmodal congruency between music and wine itself has any overarching influence on the wine drinking experience.

## Introduction

Wine writers often describe wines in terms of music. For instance, the famous British wine critic Hugh Johnson once described Robert Mondavi’s Reserve Cabernets as “Duke Ellington numbers” (see Spence, 2011 for a review). But is there any scientific basis to the oft-cited connection between music and wine, and if so, can matching music really change the wine drinking experience? The first studies to investigate the matching of sound and taste (in alcoholic beverages) were conducted by Holt-Hansen (1968, 1976). He reported that participants (*N* < 20) consistently matched two types of Carlsberg beer (Carlsberg regular and Elephant Beer) to pure tones of different frequencies (the former was placed at 510–520 Hz, whereas the latter was matched with tones in the 640–670 Hz range). What is more, drinking the beer while listening to the matching tone led to higher pleasantness ratings for certain of the participants (Holt-Hansen, 1976).

More recently, North (2012) designed a study in which the participants were assigned to one of eight conditions consisting of a unique wine–music pairing. The participants listened to a piece of music with one of the following characteristics—powerful and heavy, subtle and refined, zingy and refreshing, or mellow and soft^1^—while tasting a glass of red or white wine. They then had to rate the wine in terms of how much it reflected the same characteristics (powerful and heavy, subtle and refined, etc.). The participants (who, as university students, may not be an especially representative group of participants; see Henrich, Heine, & Norenzayan, 2010, on this point) rated the wine higher on those attributes that matched the music that they had been listening to. So, for example, the participants tended to rate the wine as more powerful and heavy while listening to music that had been categorized as powerful and heavy, than did other participants who rated the wine without music.

Meanwhile, Spence, Richards, Kjellin, Huhnt, and Daskal (2013) looked more specifically at the problem of matching music to a selection of fine wines. In the initial stages of this study, these researchers assessed how well their participants considered that a set of eight pieces of classical music matched a predetermined set of four wines (chosen to display quite different characteristics) and extracted one particularly well-matched piece of music for each wine. Next, they compared the ratings of those wines that were tasted while listening to the aforementioned matching music versus the ratings of the same wine when tasted in silence. Overall, the participants rated the wines as tasting significantly sweeter, and more liked, when tasted while listening to the putatively congruent music.

As yet, however, researchers have not examined whether there are any overarching effects on sensory ratings with congruent or incongruent music that go beyond the influence of a single piece of music on a single glass (i.e., type) of wine. (Note that North, 2012, demonstrated that the same piece of music can have the same effect on different wines, but did not assess whether the pairing of music and wine was itself congruent or incongruent.) For instance, according to the theory of processing fluency, one might predict that congruent music and food would lead to increased ratings of food pleasantness (see Labroo, Dhar, & Schwartz, 2008; Winkielman, Schwarz, Fazendeiro, & Reber, 2003); When the music and food are congruent, participants can perhaps more easily evaluate the sensory properties of the food, and consequently may find it more pleasant. By the same token, eating food while listening to incongruent music should lead to reduced pleasantness ratings.

To explicitly assess the role of music congruency on wine ratings, the present study was designed such that half of the participants listened to putatively more congruent music while tasting two different wines, while the other participants listened to music that was putatively less congruent (or possibly even incongruent).

It is worth noting that in the field of sound-odor correspondences research, the studies that have been published to date have demonstrated that odors tend to be rated as more pleasant while people are listening to congruent sounds than while listening to incongruent sounds (e.g., Seo & Hummel, 2011; Seo, Lohse, Luckett, & Hummel, 2014). Aroma-sound congruency in Seo and Hummel’s study involved the aroma of a food item (either coffee or potato chips) and the sound associated with consuming the same food (someone sipping a cup of coffee, or munching on potato chips). Meanwhile, Seo et al. used the aromas of food items and musical excerpts commonly associated with those sounds (cinnamon aroma with Christmas carols or coffee aromas with a coffee advertizing jingle). Admittedly, these examples of congruence and incongruence are much more literal, and commonly encountered in everyday life, than the more abstract pairings of music with wine.

The majority of the studies that have been published to date have assessed the perceptual effects of sound on taste perception (i.e., on specifically gustatory attributes; Crisinel et al., 2012; Spence et al., 2013; Spence, Velasco, & Knoeferle, 2014; Spence, Velasco, Vanne, & Hopia, 2014; Velasco, Jones, King, & Spence, 2013) utilized a within-participants experimental design, sometimes with the participants being aware that the taste stimuli were actually the same in the different auditory conditions (see Spence et al., 2013; Spence, Velasco, & Knoeferle, 2014; Spence, Velasco, Vanne, et al., 2014; Velasco et al., 2013). To avoid any possibility that the participants might come to realize, or believe, that they were sampling the same drink across different conditions, and hence simply give all of the drinks the same rating (presumably resulting in a null effect, or at least a reduced effect, of one’s auditory manipulation), the present study used a crossover design in which each participant rated two distinctively different wines. Furthermore, we measured a number of attributes commonly assessed in wine (Fielden, 2009), including the length of flavor, the richness, and the complexity. As music is temporal in nature, we were particularly interested in seeing whether the tempo of the music would affect the perceived length of flavor remaining in the mouth after swallowing.

## Methods and Materials

### Participants

From the 80 participants who attended a Cheesemas event held at Somerville College, Oxford University on November 28, 2014, 64 participants returned their rating sheets to the experimenters after the event. Because the experiment was conducted at a public event, the participants did not sign a standard consent form; however, the purpose of the experiment and procedure was clearly explained to them. The participants were also informed that they did not need to complete the questionnaire should they not want to, and that they could stop responding at any stage. Information concerning age and gender was not collected, although only those over 18 years of age were eligible to attend the event. The experiment was approved by the Central University Research Ethics Committee of Oxford University.

### Gustatory Stimuli

Two wines were chosen for the event. The white wine was a Marcel Martin Sauvignon Blanc 2013, from the Loire Valley in France. It has grass, citrus and gooseberry notes, light body, and high acidity. The red wine was a Para Dos Malbec 2013, from Mendoza, Argentina. It has black fruit, oak and vanilla notes, medium body, medium acidity, and soft tannins. Note that the wines were selected to be very different from one another, in order to facilitate the musical matching.

### Auditory Stimuli

Two pieces of classical music were performed by Lucia Brighenti and Irene Ortega Albaladejo from the Royal Academy of Music, London. The first was Debussy’s *Jardin Sous la Pluie*, at roughly 150 beats per minute, a virtuosic piano solo with many fast passages in a high pitch range. This piece was chosen to match the white wine, since high tempo and pitch have been shown to be associated with a sour taste and citrus flavors (Bronner, Frieler, Bruhn, Hirt, & Piper, 2012; Mesz, Trevisan, & Sigman, 2011). The second piece of music was Rachmaninoff’s *Vocalise*, a piano and cello duet played in a slow tempo (roughly 80 beats per minute). This piece was chosen to match the red wine, since legato articulation and a consonant melody have both been shown in prior research to be matched with sweet tastes and full body (Bronner et al., 2012; Mesz et al., 2011).

### Procedure

**Figure 1.**
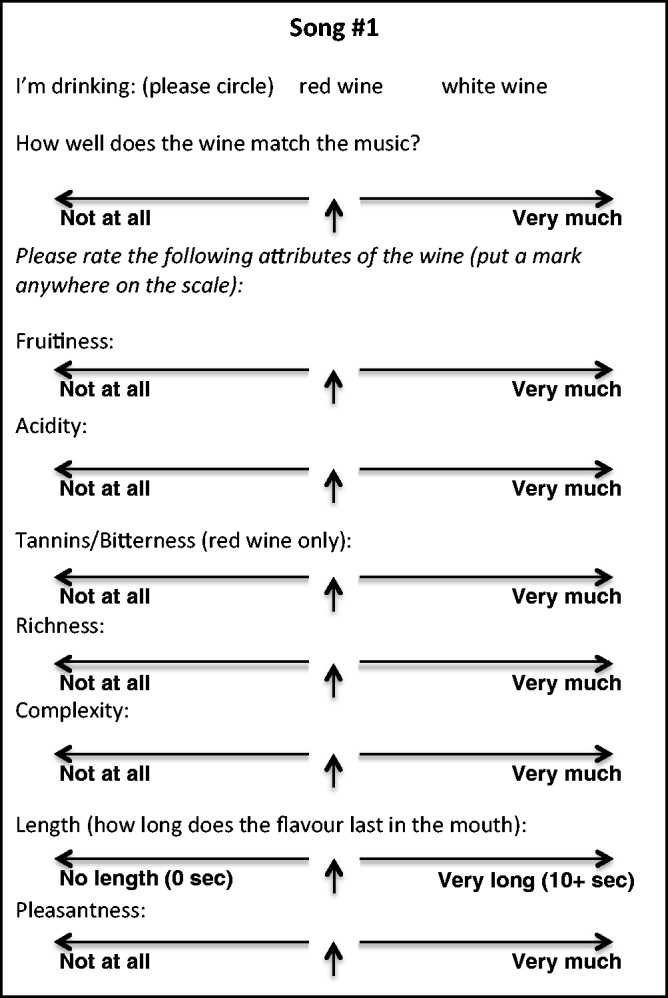
The wine rating sheet for one of the music conditions in the main experiment.

Each participant was given a glass of the white and red wine. The musicians performed the Debussy piece first, followed by the Rachmaninoff.^2^ One group of participants was instructed to taste the white wine while listening to Debussy and the red wine while listening to Rachmaninoff (therefore always tasting the wine with putatively better matching music), the other group tasted the red wine while listening to Debussy and the white wine while listening to Rachmaninoff (therefore always tasting the wine with the music that matched less well). For each song or wine pairing, the participants were instructed to fill out a rating form with scales regarding how well the wine matched the music, the fruitiness, acidity, tannins, richness, complexity, length of flavor in the mouth, and pleasantness of the wine. Each scale was 100 mm long, with the midpoint labeled, and participants had to make their ratings by marking a position along the scale (see Figure 1).

## Results

**Figure 2.**
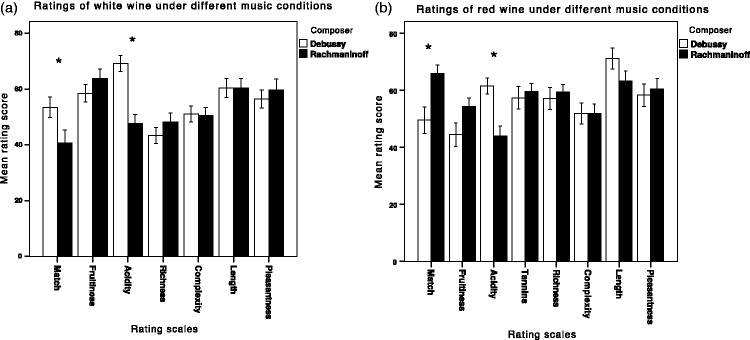
Mean rating scores of wine–music match, fruitiness, acidity, richness, complexity, length, and pleasantness for the white wine (a) and red wine (b), under both music conditions in the main experiment. Note that each scale was 100 mm long, with 0 corresponding to the midpoint of the scale. Scale ratings for all the scales were converted from the range of −50 mm to 50 mm to a score of 0 to 100, as shown on the y-axis. The error bars represent the standard error of the means. Asterisks mark significant differences (*p* < .05) between music conditions.

There were 35 participants in the putatively matching group (Group 1), who tasted the white wine while listening to Debussy and the red wine while listening to Rachmaninoff, and 29 participants in the mismatching group (Group 2). The latter tasted the red wine with Debussy and the white wine with Rachmaninoff. Scale ratings for all the scales were converted from the range of −50 mm to 50 mm to a score of 0 to 100. To compare the effect of different musical conditions on the same wine, independent samples *t* tests were performed on the ratings made by the participants from both groups for the same wine (see Figure 2(a) and (b)). In addition, mixed measures analyses of variances were performed in order to assess any overall effect of the music and music–wine congruence (and interactions thereof) on the ratings of each attribute.

For the white wine, the participants rated the Debussy (*M* = 53.47, *SD* = 21.10) as being a significantly better match than the Rachmaninoff (*M* = 40.58, *SD* = 25.90), *t*(61) = 2.18, *p* = .033. In addition, the white wine was rated as significantly more acidic while listening to Debussy (*M* = 69.20, *SD* = 16.79) than while listening to Rachmaninoff (*M* = 47.69, *SD* = 17.50), *t*(62) = 5.01, *p* < .001. However, no significant differences were observed in the ratings of fruitiness, richness, complexity, length, or pleasantness (see Figure 2(a)).

For the red wine, as expected, an independent samples *t* test revealed that the participants rated the Rachmaninoff (*M* = 65.88, *SD* = 17.60) as a significantly better match for the red wine than the Debussy (*M* = 49.48, *SD* = 25.36), *t*(48.7) = −2.93, *p* = .005. Additionally, the red wine was rated as significantly less acidic while listening to Rachmaninoff (*M* = 44.03, *SD* = 19.70) than while listening to Debussy (*M* = 61.45, *SD* = 22.16), *t*(62) = 3.88, *p* < .01. There were no significant differences in the ratings of fruitiness, tannins, richness, complexity, length, or pleasantness (see Figure 2(b)). That said, a borderline significant trend toward a difference in fruitiness is perhaps worth noting, with the red wine being rated as more fruity while listening to Rachmaninoff (*M* = 54.31, *SD* = 18.06) than while listening to Debussy (*M* = 44.49, *SD* = 22.16), *t*(53.86) = −1.93, *p* = .059).

Pearson correlations between the different rating attributes were examined (see Table 1). A significant positive correlation was observed between pleasantness and wine–music match (*r*_126_ = .175, *p* < .05), suggesting that people associate increasing congruency between wine and music with increasing pleasantness. Significant positive correlations were also observed between pleasantness and fruitiness, richness, complexity, and length; between length and both richness and complexity; between complexity and richness; between richness and acidity. In addition, there was a significant negative correlation between richness and acidity. The positive correlations between length, richness, and complexity can all perhaps be explained by the fact that these terms are all used as indicators of quality in the world of wine (e.g., see Fielden, 2009).

**Table 1.** Pearson Correlation Coefficients Between Wine Ratings for Both Red and White Wines.

	Match	Fruitiness	Acidity	Richness	Complexity	Length	Pleasantness
Match	1	.162	−.130	.193*	.121	.168	.175*
Fruitiness		1	−.072	.015	.109	.139	.247**
Acidity			1	−.231**	−.150	.007	−.123
Richness				1	.562**	.333**	.460**
Complexity					1	.390**	.428**
Length						1	.433**
Pleasantness							1

* indicates significant correlations at *p* < .05, **indicates significant correlations at *p* < .01.

Further data analysis was conducted via a two-way mixed measures analysis of variances, with music (Debussy vs. Rachmaninoff) as the within-participants factor and congruence (whether the music matched the wine or not) as the between-participants factor, for the attributes of fruitiness, acidity, richness, complexity, length, and pleasantness. (Tannin was not considered since it was only applicable to the red wine.) We examined the main effects of music and wine–music congruence as well as any interaction effects between music and wine–music congruence.

### Fruitiness

The analysis revealed a significant main effect of music on fruitiness ratings (*F*(1, 62) = 5.684, *p* = .020, $\eta_{\text{partial}}^{2}$ = .084). Specifically, the wine tasted while listening to Rachmaninoff (*M* = 59.05, *SD* = 2.29) was rated as significantly more fruity than when listening to Debussy (*M* = 51.47, *SD* = 2.55), regardless of the type of wine (white or red). In addition, in both music conditions, the white wine was rated as significantly more fruity than the red wine (*p* = .008 while listening to Debussy, *p* = .043 while listening to Rachmaninoff).

### Acidity

There was a significant main effect of music on ratings of acidity (*F*(1, 62) = 40.543, *p* < .001, $\eta_{\text{partial}}^{2}$ = .395). Specifically, the wine tasted while listening to Debussy (*M* = 65.32, *SD* = 2.03) was rated as significantly more acidic than the wine tasted while listening to Rachmaninoff (*M* = 45.86, *SD* = 2.35), regardless of the type of wine (red or white) that was being tasted.

### Richness

There was a significant interaction between music and music–wine congruence, *F*(1, 62) = 18.697, *p* < .001, $\eta_{\text{partial}}^{2}$ = .232. In both music conditions, the red wine was rated as richer than the white (*p* = .005 while listening to Debussy, *p* = .008 while listening to Rachmaninoff).

### Length

There was a significant interaction between music and music–wine congruence, *F*(1, 62) = 4.075, *p* = .048, $\eta_{\text{partial}}^{2}$ = .062. Posthoc analysis with Bonferroni corrections revealed that during the performance of Debussy, the length of the red wine was rated as significantly longer than the white wine (*p* = .036).

### Complexity, Pleasantness

None of the factors were significant for complexity or pleasantness ratings.

Overall, there were no significant differences in the ratings between the group which listened to more congruent music while drinking wine and the group which listened to less congruent music while drinking wine, except for the rating of how much the atmosphere matched the wine.

## Discussion

The results of the present study clearly demonstrate that the participants rated specific pieces of music as constituting a better match for each wine, and that the music exerted a significant effect on the perceived acidity and fruitiness of the wine. The participants’ ratings of wine–music matching verified our musical preselections, thus supplying additional evidence in support of Spence et al.’s (2013) recent findings that social drinkers often concur when it comes to matching wines to specific pieces of music. Just as for the audiovisual correspondences that have been observed between music and color (Palmer, Schloss, Xu, & Prado-León, 2013), one could, perhaps, think of this as a crossmodal correspondence between music and wine.

For each wine, there were significant differences between ratings of fruitiness and acidity under the two music conditions. Both red and white wines tasted while listening to the Rachmaninoff piece were reported to be significantly fruitier than when tasted while listening to the Debussy piece. In addition, ratings of acidity were significantly higher (on average by 20%) for both wines while Debussy was played than while the participants listened to Rachmaninoff (see Table 2 for a summary of the results from all of the music and wine studies that have been published to date).

**Table 2.** Summary of Findings From Music and Wine Studies to Date.

Study	Music	Effect on wine	Matching wine
North, 2012	*Carmina Burana*—Orff	Powerful and heavy	
	*Waltz of the Flowers* (from *The Nutcracker)*	Subtle and refined	
	*Just Can’t Get Enough*—Nouvelle Vague	Zingy and refreshing	
	*Slow Breakdown*—Michael Brook	Mellow and soft	
Spence et al., 2013	Mozart’s Flute Quartet No. 1 in D major, K 285—Movement 1	More enjoyable with music than silence	Domaine Didier Dagnueneau, Pouilly Fumé Silex 2010
	Ravel’s String Quartet in F major—Movement 1		Domaine Ponsot, Clos de la Roche 2009
	Tchaikovsky’s String Quartet No 1 in D major—Movement 2	More enjoyable with music than silence	Château Margaux 2004
Spence, Velasco, & Knoeferle, 2014	Mozart’s Flute Quartet No. 1 in D major, K 285—Movement 1	More enjoyable than *Suvitunnelma*	Tattinger Brut Réserve
	Viljami Nittykoki’s *Suvitunnelma*		Chateau Carsin Cuvée Noire 2010
Present study	Debussy’s *Jardins Sous la Pluie*	Higher acidity than *Vocalise*	Marcel Martin Sauvignon Blanc 2013
	Rachmaninoff’s *Vocalise*	Higher fruitiness than *Jardins Sous la Pluie*	Para Dos Malbec 2013

Individual rating correlations revealed that there was a significant association between music or wine matching and pleasantness. This supports the hypothesis outlined in the introduction that wines would taste more pleasant while listening to music that is congruent with the wine. However, the positive correlation between music or wine congruency and wine pleasantness in the present study is a weak one (*r*_126_ = .175, *p* < .05), in comparison to the correlations reported in Seo and Hummel’s (2011) study between sound or odor congruency and odor pleasantness (*r*_88_ = .41, *p* < .001). It is worth noting that while the congruent pairings from Seo and Hummel (2011) are likely to be commonly encountered in everyday life (e.g., coffee aroma and the sound of drinking coffee or cinnamon aromas and Christmas music), none of the participants in the present study are likely to have consciously encountered the same wine or music combinations previously. Congruence based on statistical encounters such as those from Seo and Hummel (2011) may be more strongly formed, and thus have stronger effects on processing fluency (Labroo et al., 2008; Winkielman et al., 2003), and hence pleasantness ratings.

For both wines, the participants gave higher average pleasantness ratings (although not significantly so) while listening to the Rachmaninoff piece. Perhaps this was because they enjoyed the Rachmaninoff selection more than the Debussy selection, although this cannot be verified since ratings of music pleasantness were not collected at the event.^3^ Here, it is also worth noting that in Spence, et al.'s (2014) recent study, where each wine was rated while listening to two pieces of music, one by Mozart and one by Finnish composer Niittykoski, the participants uniformly ranked the wine tasted while listening to Mozart as more pleasant, regardless of how well it matched the specific wine.

In hindsight, another piece of information that might have been helpful to collect from the participants in the present study was their wine tasting expertise. For, according to Deliza and MacFie (1996), those individuals who are less confident in their sensory abilities generally tend to be influenced more by peripheral information surrounding what they are evaluating. As wine can be considered as a notably complex flavor stimulus (e.g., Smith, 2007), one might have expected the participants’ tasting expertise to play a role in how much the music modified their perception of the wine. According to the prediction outlined here, those participants who are experienced wine tasters, and hence who are presumably more confident in their ratings of wine, may perhaps be less influenced by the sound (or any other product-extrinsic) stimuli.

On a related note, ratings involving more specific wine terminology—such as length, complexity, and richness—did not differ significantly between the two musical conditions. In contrast, acidity and fruitiness, which were significantly different under different musical conditions, are terms that are certainly more approachable, and used on a more day-to-day basis by the social drinker. It is possible that wine experts, who have more experience dealing with wine-specific terms than the casual social drinker, would give more consistent ratings.

Furthermore, the lack of any difference on ratings of the wine’s richness may be explained by background distractions during the experiment. First, it is important to note that while tasting dry wines, the perception of richness is based on the alcohol content (Fielden, 2009). In two studies looking at the effect of auditory distraction on the perception of alcohol, Stafford, Agobiani, and Fernandes (2013) and Stafford, Fernandes, and Agobiani (2012) found that, when the distractor conditions involved music, shadowing (listening to and repeating news stories), and music plus shadowing, it was the latter condition that resulted in impaired discrimination of alcohol strength. Since the participants in the present study were free to converse during the musical performance, it is possible that setting inadvertently mimicked the music and shadowing condition in Stafford et al.’s study, thus lessening the participants’ ability to judge the alcohol content of the wine (and thereby its richness). Furthermore, the live performance nature of the music possibly demanded more attention from the participants than prerecorded music played back over headphones, so it could have additionally distracted the participants and thus impacted their ability to distinguish alcohol strength.

The Rachmaninoff piece was played at a slower tempo (roughly 150 beats per minute) than the piece by Debussy (roughly 80 beats per minute). That said, the music did not have a significant effect on the ratings of the length of the wine. This did not support our hypothesis that longer musical phrases, as a result of slower tempo, might be associated with longer perceived length of the wine.^4^ Neither were our results obviously in line with Droit-Volet, Ramos, Bueno, and Bigand’s (2013) finding that music with a faster tempo leads to longer perceived time duration. As mentioned above, the length of flavor remaining in the mouth after swallowing (unlike acidity or fruitiness) is a wine-specific term that social drinkers are not likely to think about on a daily basis, therefore internal variation in length ratings is likely high. Further studies with expert participants or within-participant designs will be needed in order to untangle the question of music and wine length perception.

Nevertheless, significant differences in fruitiness and acidity ratings were observed; specifically, the Debussy piece was associated with high acidity and low fruitiness ratings, whereas the Rachmaninoff piece was associated with low acidity and high fruitiness ratings. How could these surprising associations between music and taste or flavor possibly be explained? Spence and Deroy (2013) put forward two possible mechanisms regarding how what we hear might influence what we taste or at least what we report tasting. The first mechanism works by linguistic or conceptual matching, whereby the matching sound and taste share a common descriptor (Deroy, Crisinel, & Spence, 2013; Spence & Deroy, 2013). For instance, in North’s (2012) study, hearing music that was “powerful and heavy” also increased the “powerful and heavy” rating of the wine that the participants happened to be tasting at the time. In contrast to North’s results, the different musical conditions in the present study did not result in different wine ratings on metaphorical scales such as *richness* and *complexity*, which could be used to describe both music and wine.^5^

A second mechanism proposed by Spence and Deroy (2013) is through the possibly low-level influences of crossmodal correspondences on perception. It has been shown previously that crossmodal correspondences, a notion that captures people’s tendency to match attributes from stimuli in different senses, can lead to behavioral effects that influence participants’ performance on a variety of detection and multisensory integration tasks (Deroy et al., 2013; Spence & Deroy, 2013). Crossmodal correspondences can be explained by associative learning, arising from statistical cooccurrences in the environment, or by amodal, mediated, and transitive mappings across modalities (Connolly, 2014; Deroy et al., 2013). The association that was observed in the present study between the Debussy piece and acidity could possibly be explained via the crossmodal correspondence between high pitch and sourness (Mesz et al., 2011) or between fast tempo and sourness (Bronner et al., 2012). Similarly, the association between the Rachmaninoff piece and fruitiness could be explained by a correspondence between legato articulation and sweetness (Bronner et al., 2012; Mesz et al., 2011) or between slow tempo and sweetness (Bronner et al., 2012), under the transitive property that sweetness corresponds with fruitiness as a result of associative learning.^6^

Alternatively, however, the associations between music and flavor documented in the present study could also result from their emotional similarities (for the hedonic matching hypothesis, see Deroy et al., 2013; Knöferle & Spence, 2012). For instance, it is possible that the Rachmaninoff piece was associated with fruitiness because the participants found both to be pleasant, whereas the Debussy piece was associated with acidity because they were both rated as less pleasant. Furthermore, in terms of the psychoacoustical roughness of the two pieces, more dissonant chords occur in Debussy than in Rachmaninoff. As dissonance is associated with unpleasantness, this further supports the hypothesis that participants may have found the Debussy piece more unpleasant than the Rachmaninoff. More encouragingly, studies on crossmodal correspondences have already shown the role of emotion in mediating correspondences between color and music (Barbiere, Vidal, & Zellner, 2007; Bresin, 2005; Palmer et al., 2013) and between color and aroma (e.g., Schifferstein & Tanudjaja, 2004).

On the subject of color, one needs to consider that the participants were given the red and white wines in clear glasses. Therefore, it is likely that the color of the wine itself may have entered into the crossmodal matching process (to be fair, though, even if the participants were given black tasting glasses, they could still have imagined the color of the wine as they tasted). For instance, light colors have been shown to be associated with fast tempo and dark colors to be associated with slow tempo (Palmer et al., 2013). Perhaps the white wine was matched with faster Debussy piece based on its pale straw color, and the red wine was matched with slower Rachmaninoff piece based on its deep ruby hue.

Finally, we cannot rule out order effects for these observed associations between music and flavors. Due to the public nature of the event, we could not counterbalance the music conditions across participants. The Debussy piece was performed first, then the Rachmaninoff. Therefore, it is possible that perception of acidity decreases with exposure (hence the Debussy piece, which played first, was associated with higher acidity than the Rachmaninoff piece). On the other hand, perception of fruitiness might increase with time (hence perhaps explaining why the Rachmaninoff piece was associated with rated as more fruity than the Debussy piece). Further studies with counterbalanced order of music presentation will be needed to examine this possible explanation.^7^

In general, the results of the present study demonstrate the existence of crossmodal matches between music and wine as well as the impact of music on modulating the wine drinking experience. Further testing will however be needed to reveal the specific mechanisms behind these associations between music and taste or flavors.

## Notes

1 The musical attributes were verified in a pretest in which participants (*N* = 5) had to match the four pieces of music to the four sets of adjectives. All of the assessments matched the expectations of the experimenters.

2 Due to the nature of the public event, we were unable to counterbalance the order in which the two pieces of music were presented.

3 In a separate online experiment, 14 participants rated the two pieces of music on a 10-point scale in terms of their pleasantness, complexity, and richness. Mean pleasantness ratings for the Rachmaninoff piece were somewhat higher than for the Debussy piece, but this difference was not significant (*M* = 7.43, *SD* = 1.22; *M* = 6.79, *SD* = 2.19, *t*(13) = 0.93, *p* = .370).

4 A small follow-up study (*N* = 23) was conducted with the music condition counterbalanced across participants (though this time the participants listened to prerecorded versions of the two musical selections). A significant main effect of music on length was observed, *F*(1, 19) = 5.96, *p* = .025, η^2 ^= .239. That is, those wines tasted while listening to Rachmaninoff (*M* = 6.44 s, *SD* = 0.35) were rated as significantly longer than when listening to Debussy (*M* = 4.99 s, *SD* = 0.55).

5 More tellingly, participants in a separate online study (*N* = 14) did, in fact, rate the Debussy piece as significantly more complex than the Rachmaninoff piece (on a 10-point scale, *M* = 8.00, *SD* = 1.41; *M* = 5.71, *SD* = 2.02, *t*(13) = 8.60, *p* < .01), even though no differences in complexity ratings of the wines were observed in the present study.

6 A study by Kontukoski et al. (2015) has recently shown that when asked to mix juices to match with certain pieces of music, people mix sweeter drinks when listening to sweet music and drinks that are more sour when listening to sour music. The sweet music in question had slow tempo and consonant harmonics. The sour music in question had very high pitch and fast tempo.

7 We subsequently conducted a small follow-up study (*N* = 23) with counterbalanced music conditions. We found a near-significant main effect of music on fruitiness ratings, as in the original study, *F*(1, 19) = 3.289, *p* = .086, η^2 ^= .148. Specifically, the wine tasted while listening to Rachmaninoff (*M* = 67.14, *SD* = 2.33) was rated as more fruity than when listening to Debussy (*M* = 56.550, *SD* = 4.34) regardless of the type of wine. We did not, however, observe any effect of music on ratings of acidity. This might imply that Rachmaninoff somehow increases fruitiness ratings over and above any order effects, whereas it has a less robust effect on acidity.
